# Lipome du corps calleux révélé par une crise convulsive: à propos d’un cas

**DOI:** 10.11604/pamj.2020.35.101.20440

**Published:** 2020-04-07

**Authors:** Francois Kouda, Souley Abdoulaziz, Amina Alaoui, Haloua Meriem, Alami Badreeddine, Youssef Lamrani, Mustapha Maaroufi, Meyem Boubbou

**Affiliations:** 1 Service de Radiologie Mère-Enfant, CHU Hassan II, Fès, Maroc; 2 Faculté de Médecine et de Pharmacie, Université Sidi Mohamed Ben Abdellah, Fès, Maroc

**Keywords:** Corps calleux, crises convulsives, lipome, Corpus callosum, seizures, lipoma

## Abstract

Le lipome du corps calleux est une lésion congénitale bénigne très rare, qui peut être isolée ou associée à des degrés divers de dysgénésie du corps calleux. Il peut être asymptomatique ou se révélé par des signes non spécifiques tels que des crises épileptiques, des céphalées, de déficit neurologique ou de démences. L'imagerie en coupe fait le diagnostic aisément. Nous rapportons le cas d'un adolescent de 18 ans qui a présenté une crise convulsive chez qui l'imagerie a révélé un lipome du corps calleux.

## Abstract (en)

The lipoma of the corpus callosum is a very rare benign congenital lesion, which can be isolated or associated with varying degrees of dysgenesis of the corpus callosum. It can be asymptomatic or revealed by nonspecific signs such as epileptic seizures, headache, neurological deficit or dementia. Sectional imaging makes diagnosis easily. We report the case of an 18-year-old boy who had a seizure in which imaging revealed a lipoma of the corpus callosum.

## Introduction

Les lipomes intracrâniens (LIC) sont considérés comme des lésions congénitales malformatives. Ils sont très rares et représentent moins de 0,1% des tumeurs intracrâniennes [1, 2]. Ils sont constitués de cellules adipeuses normales, anatomiquement déplacées, donc considérées comme des hétérotopies et non comme des tumeurs [3]. Ils s'observent classiquement sur la ligne médiane, et notamment au niveau du corps calleux accaparant à lui seul 90% des LIC [4]. Toutefois ces lésions ne représentent que 5% des tumeurs du corps calleux [1]. Ils peuvent être associés à degrés divers à d'autres malformations congénitales telles que l'agénésie ou dysgénésie du corps calleux. Sur le plan clinique, ils sont asymptomatiques dans la majorité des cas. Sinon ils peuvent se manifester par des céphalées ou des crises convulsives [4].

## Patient et observation

**Figure 1:**
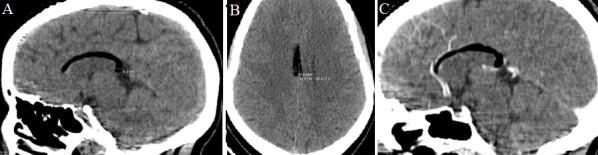
scanner cérébral en coupe sagittale sans injection (A); coupe axiale (B); coupe sagittale après injection (C): formation moulant le corps calleux bien limitée, de forme curviligne et de densité graisseuse sans prise de contraste

**Figure 2:**
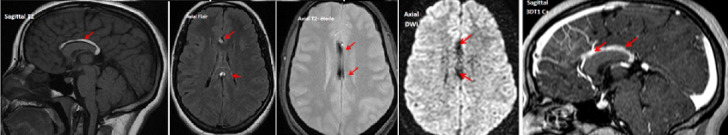
IRM encéphalique: formation curviligme (flèche rouge), moulant le corps calleux, de forme curviligne, décrite en hypersignal T1, T2 Flair, asignal T2-étoile, non restrictive en diffusion et non rehaussée après contraste en rapport avec un lipome du corps calleux

Il s'agit d'un adolescent de 18 ans sans antécédents pathologiques notables, qui a présenté un épisode de crise convulsive pour lequel il a bénéficié d'un scanner cérébral qui a révélé la présence d'une formation graisseuse, bien limitée, longeant le corps calleux mesurant 6 mm d'épaisseur (Figure 1). Pour une meilleure caractérisation, une imagerie par résonance magnétique (IRM) cérébrale a été réalisée et a montrée une formation de la ligne médiane, qui apparaît en hyper signal T1, T2 et Flair, asignal T2*, sans traduction sur la séquence de diffusion et non rehaussé après contraste (Figure 2). Le diagnostic du lipome du corps calleux a été retenu.

## Discussion

Les lipomes intra-crâniens (LIC), encore appelés hamartomes lipomateux, constituent une entité nosologique rare représentant moins de 0,1% des néoformations intracrâniennes [5]. Les premières descriptions de cette entité remontent en 1818 par Meckel (lipome chiasmatique) et en 1856 par Rokitansky qui a décrit un lipome du corps calleux associé à une agénésie du corps calleux [1]. Cette entité est souvent rencontrée chez la population pédiatrique et l'adulte jeune. Notre patient était aussi jeune, âgé de 18 ans. Ces lésions sont plutôt malformatives et non tumorales, constituées histologiquement de cellules graisseuses normales et seraient dues à des anomalies de différenciation avec persistance du tissu mésenchymateux de la meninx primitiva [5, 6]. Plus d'un siècle après sa première description, l'étiopathogénie précise des lipomes intracrâniens est encore discutée. Plusieurs théories comme l'hypertrophie du tissu adipeux pré-existant dans les méninges, la métaplasie du tissu conjonctif méningé, les malformations hétérotopiques d'origine dermique, et pseudo tumeur dérivée de la méninge primitive ont été avancées pour expliquer l'histogenèse de ces lésions. Actuellement, on admet que ces lipomes sont une anomalie de la différenciation de la méninge primitive persistante, qui se résorbe normalement entre la 8^e^ et la 10^e^ semaine de gestation [1]. La localisation classique des LIC est la ligne médiane dans 90% des cas, et le site le plus fréquent est la région dorsale péri calleuse [1, 5]. Toutefois, les lipomes du corps calleux ne représentent que 5% des tumeurs calleuses [3]. Ils sont souvent associés à d'autres anomalies de la différenciation des structures médianes, notamment à une hypogénésie ou une agénésie du corps calleux, qui sont retrouvées dans 90% des lipomes antérieurs et dans 30% des lipomes postérieurs [7]. Dans notre cas il n'y avait pas de malformation du corps calleux. Les autres anomalies liées à la présence des lipomes intracrâniens comprennent, une agénésie du vermis cérébelleux, des tumeurs de l'hypophyse, des schwanomes acoustiques et d'autres lipomes intracrâniens plus souvent situés dans le plexus choroïde des ventricules latéraux [1, 2, 5].

Les lipomes du corps calleux sont morphologiquement classés en deux groupes [6]; **les lipomes antérieurs:** c'est la forme tubulo-nodulaire qui sont arrondis ou lobulaires et de taille généralement supérieur à 2 cm d'épaisseur et sont généralement associés à une hypogenèsie/agénésie du corps calleux, des anomalies des lobes frontaux, calcifications, et/ou des anomalies oculaires; **les lipomes postérieurs dits curvilignes:** ils sont minces et allongés le long de la marge du corps calleux, et de taille habituellement inférieur à 1 cm d'épaisseur. Ils siègent plus en arrière sur le splénium et sont moins souvent associés à des anomalies corps calleux et/ou d'autres anomalies encéphaliques. Notre cas était de ce groupe puisque la lésion était postérieure, curviligne mesurant 6 mm, sans autre anomalie malformative associée.

Les lipomes du corps calleux isolés sont généralement asymptomatiques et sont donc découvertes fortuitement [2]. Cependant, notre patient a présenté une crise convulsive sur lipome du corps calleux isolé. Les manifestations cliniques lorsqu'elles sont présentes sont secondaires aux anomalies du tissu nerveux associées [6]. Ces manifestations sont polymorphes et aspécifiques dominées par l'épilepsie partielle qui apparait dans ce cas avant 15 ans [2, 8]. Les autres manifestations sont: les céphalées, les troubles mentaux, hémiparésie [1]. Ils peuvent aussi être responsables d'une hydrocéphalie active [6]. L'imagerie est la clé du diagnostic [1]. En prénatal le diagnostic est possible par échographie à partir de la 26^e^ semaine de gestation [7]. Les radiographies peuvent montrer des calcifications curvilignes notamment dans la variété tubulo-nodulaire. Toutes fois sa contribution reste négligeable, donc de moins en moins indiquée [1]. Au scanner, ces lipomes apparaissent comme des masses de densité graisseuse (-80 à -110 UH), qui peuvent contenir des calcifications périphériques. Des calcifications curvilignes périphériques se voient souvent dans la variété tubulo-nodulaire appelées « bracket sign » sur les images reconstruites coronales [1, 2]. L'IRM est l'examen de choix. Elle permet non seulement de caractériser l'extension du lipome, mais aussi de rechercher l'agénésie ou une dysgénésie du corps calleux fréquemment associées. Ils apparaissent en hyper signal T1 et T2, avec chute du signal sur les séquences de Fat Saturation [1, 2, 6]. Le diagnostic différentiel se discute avec les kystes et les tératomes dermoïdes, une faux du cerveau « graisseuse »: en particulier devant le type curviligne, ou une rare transformation lipomateuse de certaines tumeurs: tumeurs neuroectodermiques primitives (TNEP), épendymome, gliome [1, 2]. Aucun traitement spécifique n'est habituellement exigé. On propose un traitement symptomatique antiépileptique devant des crises d'épilepsie [1, 2, 7]. Le traitement chirurgical est rarement indiqué car il est difficile de réaliser une réduction complète qui épargne les structures vasculo-nerveuses impliquées en péri-calleuses [2].

## Conclusion

Les lipomes du corps calleux sont des entités nosologiques très rares, associée à des degrés divers aux anomalies du corps calleux. Ils sont souvent asymptomatiques dans la majorité des cas et découverts fortuitement. Les signes cliniques ainsi que le pronostic dépendent de la malformation associée. L'imagerie en coupe sur l'IRM permet de faire le diagnostic. Le traitement est symptomatique à base d'antiépileptique en cas de crises épileptiques. La chirurgie reste rarement indiquée.

## Conflits d’intérêts

Les auteurs ne déclarent aucun conflit d'intérêts.
